# Vascular injuries and complications in anterior lumbar interbody fusion: an up-to-date review

**DOI:** 10.25122/jml-2024-0345

**Published:** 2025-03

**Authors:** George Tsalimas, Athanasios Galanis, Michail Vavourakis, Evangelos Sakellariou, Dimitrios Zachariou, Iordanis Varsamos, Christos Patilas, Ioannis Kolovos, Vasilis Marougklianis, Panagiotis Karampinas, Angelos Kaspiris, Spiros Pneumaticos

**Affiliations:** 1 3^rd^ Department of Orthopaedic Surgery, National & Kapodistrian University of Athens, KAT General Hospital, Athens, Greece

**Keywords:** lumbar spine, anterior lumbar interbody fusion (ALIF), vascular injury, surgical complications, LIF, Lumbar Interbody Fusion, ALIF, Anterior Lumbar Interbody Fusion, PLIF, Posterior Lumbar Interbody Fusion, TLIF, Transforaminal Lumbar Interbody Fusion, PSF, Posterior Spinal Fusion, LL, Lumbar Lordosis, PI, Pelvic Incidence, CT, Computed Tomography, 3D CT, Three-Dimensional Computed Tomography, CTA, Computed Tomography Angiography, MRI, Magnetic Resonance Imaging, MRV, Magnetic Resonance Venography, IVC, Intravenous Catheterization, BMI, Body Mass Index, DVT, Deep Venous Thrombosis

## Abstract

Vascular injuries during anterior lumbar interbody fusion (ALIF) are reported in the existing literature with an incidence rate ranging from 1% to 24%, predominantly venous lacerations owing to branch vessel avulsions during mobilization and retraction. Arterial injuries, although less frequent, occur at an incidence of 0.45% to 1.5% and are mainly characterized by thromboses; aortic lacerations remain exceptionally rare. L4-L5 and L5-S1 are the two levels associated with the majority of vascular complications. Preoperative 3D CT angiography is paramount and a gold standard, as it illustrates the anatomic variations of the iliolumbar vein, the aorta, and the vena cava bifurcation, providing the surgeon with valuable information regarding operative trajectories. Regarding preventive measures, venous laceration, the most common vascular injury, occurs less frequently when employing nonthreaded interbody grafts such as iliac crest autograft or femoral ring allograft. Also, left iliac artery thrombosis can be decreased intraoperatively by intermittent release of retraction. Managing vascular complications includes compression for bleeding control, Trendeleburg positioning of the patient and venorrhaphy, and the employment of topical clot-forming enhancement and/or hemostatic agents. Although postoperative lower limb duplex ultrasonography can be an effective tool, magnetic resonance venography (MRV) and intravenous catheterization (IVC) remain the gold standards for diagnosing postoperative pelvic vein thrombosis in cases of iliac vein repair after anterior spine surgery. This paper aimed to highlight the incidence of major vascular injury during ALIF surgery, describe predisposing risk factors, and discuss management techniques while highlighting the requirement for more sensitive and factor-specific studies to attain a more profound understanding of the mechanism of vasculature complications during ALIF procedures.

## INTRODUCTION

**Figure 1:**
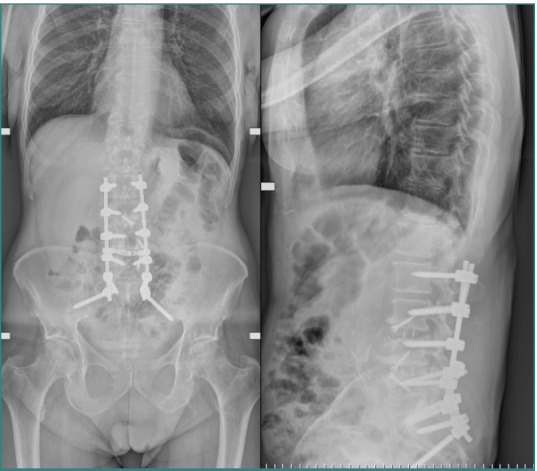
Postoperative F&P X-rays depicting posterior spinal fusion from L2 to S1 with ALIF at L4-L5 and L3-L4 levels

Anterior lumbar interbody fusion (ALIF) has evolved into a growingly prevalent approach for treating a wide range of spinal pathologies, as it offers anterior column reconstruction of the lumbar spine while facilitating restoration of normal lumbar lordosis (LL) [1-3] (Figure 1). Moreover, indirect decompression of the foramina can be acquired due to increased intervertebral height produced by the cage or graft. A more thorough understanding of spine biomechanics and the availability of grafts in various sizes have contributed to the popularity of ALIF [1-4].

Appropriate patient selection and clear indications are pivotal for good surgical outcomes in ALIF surgery. As supported by various studies in the literature, chronic low back pain is an indication of ALIF [1]. In addition, another ALIF indication is pseudarthrosis following failed dorsal lumbar fusion with or without interbody grafting, in which cases L3‒L4, L4‒L5, or L5‒S1 are ordinarily the levels of intervention. Although ALIF is not customarily utilized for neural elements decompression, owing to the nature of the surgical approach, indirect neural foramina decompression is achieved via the widening of the interpedicular space [1-3].

An enhanced understanding of sagittal balance and global alignment importance drove ALIF procedures to develop as a well-established surgical option, supported by numerous studies, such as Lafage *et al*. [2], who illustrated the concern of maintaining LL within 9 degrees of pelvic incidence (PI), which is best accomplished with large, lordotic-shaped, intervertebral lower lumbar struts.

Although ALIF is generally associated with low complication rates [3], complications can still arise during the preoperative, intraoperative, or postoperative phases. A study by McDonnell *et al*. [4], which analyzed 447 patients, reported that 11% experienced major complications, while 24% had minor complications following anterior spinal surgical approaches. The complications can derive from the surgical approach, discectomy-related factors, or injuries involving the abdominal viscera, abdominal wall, major blood vessels, ureter, and nerve plexuses. Although vascular injuries are relatively uncommon, cases have been documented in the literature, typically classified as thrombosis, occlusion, or rupture [1-5].

This narrative review aimed to examine the incidence of major vascular injuries during ALIF procedures, identify risk factors and discuss management strategies. This review also highlights the need for more comprehensive research, as the current literature on this topic remains limited.

## MATERIAL AND METHODS

The literature search was carried out using the MEDLINE/PubMed, Web of Science, and Google Scholar databases. The search included the following keywords: 'ALIF', 'anterior spine surgery', 'ALIF complications', 'ALIF vessel injury', 'ALIF vascular complications', 'spinal surgery vascular injuries', and 'spine surgery vascular injuries'. The search was restricted to English-language articles, with no limitations on publication date. Inclusion criteria encompassed clinical studies, case series, reviews, and papers reporting data relevant to the topic. Exclusion criteria included studies focusing exclusively on vascular injuries and complications related to other types of spinal surgery. Full-text articles were reviewed to identify additional relevant studies. Two authors independently screened and selected articles based on the inclusion criteria. In cases of disagreement, a third author was consulted to resolve discrepancies and make the final decision. Extracted data included author names, diagnosis, gender, publication date, patient age, study location, and type of intervention.

### Surgical approach and vasculature

The most widely employed surgical approaches are the left-sided retroperitoneal dissection, the transperitoneal open procedure, and the laparoscopic approach [5-7]. The retroperitoneal approach is preferred over transperitoneal surgery due to ileus and oedematous bowel complications associated with the latter. A general or vascular surgeon might be requisite if the spine surgeon is not sufficiently comfortable with the approach regarding the management of potential intraoperative complications and proper vessel ligation techniques for exposure of the disc spaces [8,9]. However, there is controversial evidence regarding the benefits offered by the presence of an access surgeon. This could be attributed to the variable level of spine surgeons' training, the discrepancies in healthcare systems protocols concerning the need for the participation of an access surgeon in ALIF procedures, and the fact that access surgeons broadly take part in more onerous and intricate cases [1,10]. A 2009 study by Jarrett *et al*. [11] reported no significant change in complication rates, including vascular injuries when an access surgeon was present. Nonetheless, that study was small in size, and it was a retrospective and nonrandomized research that could lead to potential confounders and featured several biases. Also, a meta-analysis by Phan *et al*. [12] suggested that the intraoperative vascular complication rates were comparatively similar regardless of whether an access surgeon participated.

In a left-sided retroperitoneal approach, the intraperitoneal contents must be carefully mobilized from the left side toward the midline, ensuring that the ureter is identified and protected. Dissection should proceed anterior to the psoas muscle to minimize the risk of genitofemoral and ilioinguinal nerve injury. Postoperative internal hernia, bowel obstruction, and incarceration have all been documented after peritoneal disruption [8,9,13]. Recognizing the correct surgical level with intraoperative radiographs is vital, as erroneous level surgeries have been reported [13,14].

**Figure 2:**
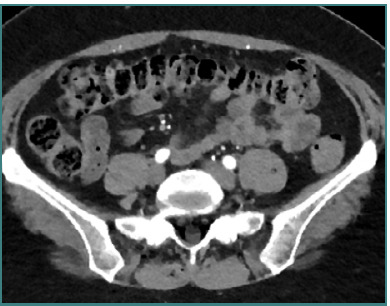
Axial view of L5-S1 level on CTA

**Figure 3:**
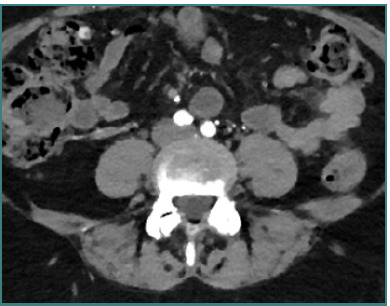
Axial view of L4-L5 level on CTA

Lumbar segmental arteries should be respected and not routinely ligated, as they provide blood supply to the vertebral body [15]. Regarding the L5‒S1 interspace (Figure 2), the surgical interspace is located between the right and left common iliac vessels with no need for vessel ligation, as opposed to the L4‒L5 interspace (Figure 3), where the left common iliac vessels cross transversely the prevertebral space and, therefore, they must be mobilized from left to right to expose the bony midline.

Additionally, the left iliolumbar vein should be ligated to achieve optimal exposure at this level, as it tethers the left common iliac vein laterally and caudally to the inferior vena cava [7]. Common iliac vein injuries exhibit a reported incidence varying from 2% to 4.5% [16-18], whilst common iliac arteries and inferior vena cava injuries are considerably rarer. For L1–L2 levels, aortic mobilization from left to right is necessary to gain bony access, with careful attention to avoiding trauma to the left renal artery. The iliac vessels must be medially mobilized at the L3–L4 disc space to ensure safe exposure [19-21]. A systematic review by Feeley *et al*. [10] analyzed vascular complications across different ALIF approaches and found no statistically significant differences in vessel injury rates based on the type of approach [10]. However, the transperitoneal approach demonstrated higher rates of retrograde ejaculation and overall complication rates.

Regarding retractor systems, most surgeons commonly use self-retaining or automatic retractors. However, there is ongoing debate about whether hand-held retraction—employed by a minority—provides superior manipulation and control of major blood vessels, such as the aorta, the superior and inferior vena cava, and the iliac arteries and veins [18-20].

### Venous and arterial vascular injuries

The incidence of vascular injuries in anterior spinal surgery varies widely across case series. These injuries, which involve the iliac vessels, vena cava, and aorta, are considered among the most severe complications, with reported incidence rates ranging from 1% to 24% in the literature [22-28]. The rate may vary depending on patients’ demographics, the number or level of lumbar spine regions to be fused, and the surgical approach.

The majority of vascular injuries are venous lacerations, primarily resulting from branch vessel avulsions during mobilization and retraction [22,24,25,29,30]. The left common iliac vein is the most frequently injured, followed by the inferior vena cava and iliolumbar vein [31]. Most venous injuries occur during the retraction of the great vessels. Other situations related to venous injury include discectomy, placement of the interbody graft, and removal of the Steinman pin, with the main mechanisms of injury being large vein tears and segmental vein avulsions [22,24,25,29,30]. Injuries to the inferior vena cava are rare. Brau *et al*., in their study, suggested that this complication was dealt with only by ligation of the inferior vena cava, and postoperatively, the patients presented edema of bilateral lower extremities [22]. However, it should be underlined that the research from Brau *et al*. [22], although a large-scale study, presents data from procedures executed between 1997 and 2002.

Even though arterial injuries are less common compared to venous, they have been documented with an incidence rate ranging from 0.45% to 1.5% [29,30,32], consisting predominately of thromboses. Aortic lacerations are particularly rare due to the greater elasticity of the abdominal aorta and iliac arteries than veins, making them less susceptible to injury during retraction. However, left iliac artery injuries have been more frequently observed, as documented by Brau *et al*. [22] and Watkins R. [23]. Similarly, Faciszewski *et al*. [32] reported aortic laceration complications at a rate of 0.08%. Due to prolonged retraction of right common iliac vessels, thrombosis of left iliac arteries can present as lower extremity pain and motor or sensory deficits and must be distinguished from nerve root irritation. To minimize this risk, manual retraction of the iliac vessels at 20-minute intervals is now standard practice [19,31]. Although this complication is mainly characterized by early onset hours after surgery, late-onset has also been reported by Goodkin *et al*. [33,34], with the left common iliac artery more frequently affected [35,36]. It should be noted that these studies, although methodologically consistent, were carried out more than 20 years ago.

The majority of vascular injuries occur in the iliac venous system during the exposure of L4-L5 and L5-S1 [25,27,37], so a compelling point is that studies concentrating on more cephalic and higher lumbar levels may demonstrate lower rates of vascular injury [19]. In terms of deep vein thrombosis (DVT) and thromboembolic events, a study by Nourian *et al*. documented that patients sustaining an intraoperative vascular injury in ALIF surgery were considerably more likely to experience postoperative thromboembolic events compared to patients who did not (36% and 5%, respectively); therefore prophylaxis may be considered in these cases. Also, it was inferred that DVT and postoperative thromboembolic complications were not correlated to anatomic variation, approach level, blood loss, and patient characteristics [38]. However, a key limitation of this study is its level of evidence (LOE 4).

### Risk factors and preoperative radiographic planning

**Figure 4:**
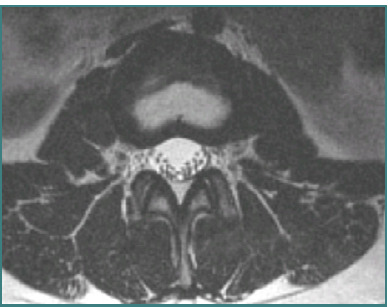
Axial view of L4-L5 level on MRI

Preoperative three-dimensional computed tomography (3D CT) angiography plays a crucial role and is considered the gold standard in cases where anatomic variations are a concern. This imaging modality allows for the identification of iliolumbar vein variations, as well as the bifurcation points of the aorta and vena cava, providing critical information for surgical planning and trajectory optimization, as proposed by Regan *et al*. [39]. Other vitally important anatomic variations may also involve the local vessels, such as left-sided or duplicated IVC, left renal vein retro-aortic course, as well as variations of the lumbosacral junction [40,41]. Contrarily, many clinicians postulate that a dedicated 3D CT angiography is not required in most cases. They propose that in some complex cases, although not always necessary, where procuring more elaborate anatomic details is essential, a CT with contrast would be ideal for delineating the vessel anatomy compared to a lumbar spine MRI (Figure 4) which is ordinarily obtained, especially when dealing with L2-L3, L3-L4 and maybe even L4-L5 levels [35-37,39-41].

Chronic infections, such as tuberculosis, tumors, or even old fractures, may trigger vasculature adhesions to the vertebral column and intervertebral disc due to ongoing inflammatory processes, prompting an increased risk of vascular injury and laceration [40]. Other predisposing factors reported in literature associated with local perivertebral tissue inflammation resulting in lack of mobility during manipulation in surrounding vessels are spondylolisthesis, anterior osteophytes, previous spinal infection, revision surgery for migrating spinal devices, as well as previous anterior spine surgery [39-41]. A recent single-center retrospective cohort study by Mortazavi *et al*. examined complication rates and risk factors associated with postoperative complications following 362 ALIF procedures. They reported that vascular complications were the third most common complication (3.6%) after surgical site infections and neurological complications (5.8% and 4.1%, respectively). Patients with a higher mean age, body mass index (BMI), and diabetes had an increased risk for vascular complications after ALIF surgery [42]. The study by Mortazavi *et al*. [42] was a single-center and retrospective study that only reported factors correlated to perioperative complications; therefore, further research is needed to determine causal associations of patients and operative factors with complication profiles. Furthermore, a 2021 study by Ho *et al*. [43] indicated that increased vertebral exposure in ALIF procedures was associated with enhanced risk of intraoperative venous injury and postoperative deep vein thrombosis (DVT). However, this study lacked data on anatomic factors, such as pelvic incidence, scoliotic deformity degree, and variant vascular anatomy. In another study conducted in 2021, Wert Jr *et al*. [44] examined the risk factors for complications during ALIF surgery exposure, indicating for the first time male sex is a risk factor for complications, including perioperative vascular injuries. In this study, venous injury was the most common complication at a 10% rate, while no arterial injury was reported. However, key limitations of this research include its retrospective design, small sample size, and statistical challenges with c-statistics and logistic regression. A recent systematic review by Feeley *et al*. [45] suggested that there was no significant difference in vascular complication rates between obese and non-obese patients undergoing ALIF surgery. However, patients with obesity had considerably increased rates of other complications, such as wound complications and reduced fusion rates. Another important risk factor for vascular complications in ALIF surgery is atherosclerosis [37,39-41]. As vessel elasticity declines, mobilization of the iliac vessels becomes more challenging, increasing the risk of vascular injury. Therefore, assiduous preoperative radiographic planning is essential. Even without visible atherosclerotic plaques, soft atherosclerotic tissues that might rupture can be present, leading to vessel occlusion and thrombosis [33-37,39-41]. Additionally, a review by Than *et al*. [46] examining complication avoidance and management in ALIF surgery suggested that venous laceration, the most common vascular injury, occurs less frequently when utilizing non-threaded interbody grafts, such as iliac crest autografts or femoral ring allografts. Additionally, the risk of left iliac artery thrombosis can be reduced by intermittently releasing retraction intraoperatively [46]. A recent single-center retrospective study by Yunga Tigre *et al*. [47] identified older age, opioid use, multilevel ALIF involving L2-L3, and multiple interbodies as statistically independent predictors of vascular injury in ALIF procedures. On the other hand, a retrospective cohort study by Ahn *et al*. found no correlation between age and vascular complications in ALIF procedures, suggesting that older age might not be an absolute contraindication for ALIF surgery [48]. When compared to other lumbar interbody fusion (LIF) techniques, ALIF has been reported to have a higher incidence of vascular injury than transforaminal lumbar interbody fusion (TLIF) (2.6% vs. 0%, respectively) [49,50]. Conversely, a systematic review by Rathbone *et al*. [51] concluded that vascular complication rates do not significantly differ between ALIF and posterior lumbar interbody fusion (PLIF). Finally, it is important to emphasize that robust data on the impact of implant type on vascular complication rates in ALIF surgery remain scarce in the current literature; thus, pertinent research is crucial in this area.

### Management of vascular injuries

Vascular injuries during ALIF procedures are of critical concern, as their complications and effective management can be lifesaving. Regarding iliac venous lacerations, initial bleeding control compression should be carried out proximally and distally to the laceration, respectively, while suction should be avoided, as it may induce venotomy extension [36,37,39-40]. However, given the practical challenges of completely avoiding suctioning during an iliac vein injury, surgeons must act swiftly and efficiently when addressing these complications [36-37, 39-40]. Kitner dissectors and sponge sticks can be employed. If feasible, the patient should be placed in the Trendelenburg position to diminish venous pressure in the lower extremities [52]. For venorrhaphy, 4-0 or 5-0 Prolene stitches are commonly used, while topical clot-forming enhancement and/or hemostatic agents can aid in preserving hemodynamic stability in incomplete repairs [52]. A literature review underscores that even minimal manipulation of the iliac venous system can trigger thrombosis or vessel occlusion. Although postoperative lower limb duplex ultrasonography can be a very functional instrument, MRV and IVC are still the gold standards for detecting postoperative pelvic vein thrombosis in cases of iliac vein repair following anterior spine surgery [53].

## CONCLUSION

With the increasing life expectancy of the global population and the growing demand for complex spine surgeries, the role of ALIF procedures continues to expand. To minimize the risk of vascular injuries and associated complications, surgeons must employ meticulous operative techniques and adhere to careful surgical principles. Comprehensive preoperative imaging and planning are pivotal for detecting potentially detrimental anatomic variations and risk factors. Additionally, calm and precise intraoperative management when vascular injuries occur is of paramount significance. Further research is necessary to provide greater clarity on the underlying mechanisms of vascular complications in ALIF surgery.

## Data Availability

All raw data are available to access should they be requested.
